# Splenial Callosal Disconnection in Right Hemianopic Patients Induces Right Visual-Spatial Neglect

**DOI:** 10.3390/brainsci12050640

**Published:** 2022-05-12

**Authors:** Francesco Tomaiuolo, Giovanni Raffa, Serena Campana, Giada Garufi, Stefano Lasaponara, Loredana Voci, Salvatore M. Cardali, Antonino Germanò, Fabrizio Doricchi, Michael Petrides

**Affiliations:** 1 Department of Clinical and Experimental Medicine, University of Messina, Piazza Pugliatti, 1, 98122 Messina, Italy; 2 Division of Neurosurgery, Department BIOMORF, University of Messina, Piazza Pugliatti, 1, 98122 Messina, Italy; graffa@unime.it (G.R.); giadagarufi@hotmail.it (G.G.); scardali@unime.it (S.M.C.); 3 Neurorehabilitation Unit, Auxilium Vitae Volterra, Via Borgo San Lazzero 5, 56048 Volterra, Italy; sera.campana@gmail.com (S.C.); l.voci@riabilitazione-volterra.it (L.V.); 4 Department of Psychology, La Sapienza University, Piazzale Aldo Moro 5, 00185 Rome, Italy; lasaponara.stefano@gmail.com (S.L.); fabrizio.doricchi@uniroma1.it (F.D.); 5 Laboratorio di Neuropsicologia dell’Attenzione, Fondazione Santa Lucia IRCCS, Via Ardeatina, 306, 00179 Rome, Italy; 6 Dipartimento di Scienze Umane, Libera Università Maria Santissima Assunta LUMSA, Via della Traspontina, 21, 00193 Rome, Italy; 7 Montreal Neurological Institute, McGill University, 3801 University Street, Montreal, QC H3A 2B4, Canada; michael.petrides@mcgill.ca

**Keywords:** neglect, neuropsychology, clinical neurology, neurosurgery, neuroanatomy

## Abstract

Posterior cerebral artery (PCA) territory infarction involving occipital cortical damage can give rise to contralateral homonymous hemianopia. Here, we report two rare cases of patients with lesions in the left hemisphere PCA territory who developed right visuo-spatial neglect. One patient suffered right hemianopia and right visuo-spatial neglect after a stroke that damaged the left primary visual cortex and the callosal splenial fibers. The other unique case is of a patient who had a brain tumor in the posterior cerebral region in the left hemisphere and initially exhibited only right hemianopia that developed into right visuo-spatial neglect after tumor resection that included the splenial fibers. These cases indicate that, as in cases with damage in the right PCA territory, lesions in the left PCA yield visuo-spatial neglect when the damage produces contralateral hemianopia and concomitant disconnection of the splenium of the corpus callosum, which interferes with the arrival of visual inputs from the intact right to the lesioned left hemisphere. These results also emphasize the necessity of sparing the splenial fibers in surgical interventions in patients who exhibit hemianopia.

## 1. Introduction

Spatial neglect is a syndrome characterized by failure to attend to and explore the side of space contralateral to unilateral brain damage. Neglect most frequently follows right hemisphere brain damage in the territory of the middle cerebral artery (MCA) that disrupts intra-hemispheric functional interactions between parietal and frontal areas, e.g., via damage to the arcuate and superior longitudinal fasciculi [1,2,3,4,5,6,7]. However, patients with right-sided neglect after left hemisphere damage have also been reported [8,9]. Neglect may also occur from lesions in the posterior cerebral artery (PCA) territory of the right [10,11,12,13] and of the left hemisphere, although in the latter case neglect is less severe [11]. Contralesional visuo-spatial neglect has been reported after neurosurgical lesion studies in the monkey [14] and in patients, in both group brain lesion [10,12] and single-case [11,13] studies. In these studies, brain damage produces contralesional hemianopia by damaging the primary visual cortex and/or its afferent connections and, in addition, visuo-spatial neglect when the damage extends to the adjacent splenium of the corpus callosum [10,11,12,13,14,15]. In such cases, parietal and frontal areas in the damaged hemisphere cannot build-up a visual-spatial representation of the contralateral space, because callosal disconnection impairs the arrival of any residual visual information from the intact hemisphere [14,15]. Recently, Sperber and colleagues [16] have examined the anatomy of spatial neglect after unilateral right hemisphere PCA stroke and concluded that left hemispatial neglect was related to lesion of the occipito-parietal region, but did not detect unilateral neglect in relation to damage of the splenial fibers per se. Thus, disruption of interhemispheric interaction via the splenial cortical fibers is not sufficient to lead to neglect. In the present study, we demonstrate that unilateral neglect can arise after damage to the splenial fibers if there is additional damage to the primary visual cortical region that deprives the hemisphere of any visual input. In the latter case, the splenial damage prevents communication between the posterior cortical regions in a brain, in which one hemisphere lacks visual input as a result of post-geniculo-calcarine tract damage.

In the present study, we report the cases of two patients with left posterior hemisphere damage who exhibited chronic right neglect restricted to the visual domain when damage to the splenial fibers was added to primary visual cortical impairment. One patient (P1) suffered right hemianopia and right visuo-spatial neglect after an ischemic stroke in the territory of the PCA, damaging the left primary visual cortex and the callosal splenial fibers. The other patient (P2) initially had right hemianopia that developed into right visuo-spatial neglect after surgical neoplasm removal that included splenial fibers in the forceps major of the corpus callosum. A third control patient (P3) developed right hemianopia without visuo-spatial neglect after surgical neoplasm removal that included the optic radiation sparing the splenial fibers of the corpus callosum. This evidence supports the critical role of the combination of splenial damage with contralesional hemianopia in the genesis of contralesional hemispatial neglect in patients with lesions in the PCA territory.

## 2. Materials and Methods

The participants provided written informed consent and this Non-Pharmacological Retrospective Observational Study has been communicated to the National Research Ethics Committee of Messina (Prot. 383-20). None of the two patients had impaired level of consciousness or muteness, and there was no history of dementia or hearing loss. Thus, the selective nature of the impairment of the patients P1 and P2 indicates that the deficit was not due to a failure in understanding the instructions. For example, the patients performed normally on the Fluff test [17] (i.e., understood the instructions), but were impaired on other tests, such as the line bisection, cancellation tasks, and the drawing task.

Patient 1 (P1): Female, 79-year-old, right-handed patient, with 12 years of education. She suffered from hypertension and dyslipidaemia and because of that, she had specific drug treatment. She arrived at the Emergency Unit in a coma (Glasgow Coma Scale [18] score 4). An Angio-CT brain scan revealed a thrombotic obstruction of the medial tract of the basilar artery that was treated with thrombus aspiration. She was sedated and supported with mechanical ventilation for 8 days, and after two failed extubation attempts, she underwent a tracheotomy. On day 16, she recovered consciousness and was transferred from the intensive care unit to the severe acquired brain lesion unit. When assessed at 117 days after the ischemic stroke, she was able to comprehend and produce simple verbal sentences and was oriented in space, but not oriented in time. She moved her eyes only from the center to the left side and had right spatial dyslexia, i.e., she correctly read only the left side of words and sentences. In addition, she had dysgraphia. She was unaware of her visual disabilities and her right spatial neglect. Humphrey perimetry testing revealed complete right hemianopia.

Patient 2 (P2): Male, 75-year-old, right-handed patient, with 5 years of education. He was in good health, suffering from hypertension. Recently experienced infrequent episodes of slurred speech with severe difficulties in naming objects and/or memory deficit that usually resolved spontaneously after a few minutes. Such episodes were considered the expression of focal seizures and the patient was treated with levetiracetam 500 mg BID. The Electro Encephalogram was not performed before surgery. He was oriented in space and time (except for the day of the month). Clinical examination revealed anomia, agraphia, acalculia, and alexia; he was not able to read single letters, but he was able to read and write single-digit numbers, and he could sign. No neglect signs were present. Humphrey perimetric testing of the visual field revealed complete right hemianopia. He subsequently underwent microsurgical resection of a glioblastoma (WHO grade 4) through a left parieto-occipital craniotomy, following an almost-perpendicular direction to the interhemispheric fissure. Clinical examination on the second day after surgery demonstrated unchanged cognitive abilities, but now the patient exhibited, in addition, right visuo-spatial neglect. No further seizure episodes were reported during the follow-up. The visuo-spatial neglect was still present when the patient had a neglect assessment administered 30 days after surgery.

Patient 3 (P3): This was the control patient who was a male, 37-year-old, right-handed person, with 8 years of education. He had previous allergic reactions to antibiotics and also had a previous surgery for an inguinal hernia. He was admitted in the Neurosurgery Department for frequent episodes of altered vision. The clinical evaluation revealed a right superior quadrantanopia without any motor or sensory deficit nor a cognitive deficit (Montreal Cognitive Assessment [19]: scored 30/30) and, in particular, no neglect signs. The patient underwent an ophthalmologic evaluation, which confirmed the right superior quadrantanopia. The MRI brain scan revealed the presence of a lesion in the deep white matter of the posterior temporal and parietal-occipital region next to the posterior horn of the left lateral ventricle. The patient underwent a right parieto-occipital craniotomy following an almost-parallel direction to the interhemispheric fissure with a gross total resection of the lesion. The microscopic pathological evaluation documented a low-grade glioma (WHO grade 2). Clinical evaluation on the 1st day after surgery demonstrated a complete right hemianopia and unchanged cognitive abilities. Humphrey perimetry testing 7 days after surgery showed a right hemianopia.

### 2.1. Neuroanatomical Processing

**Figure 1:**
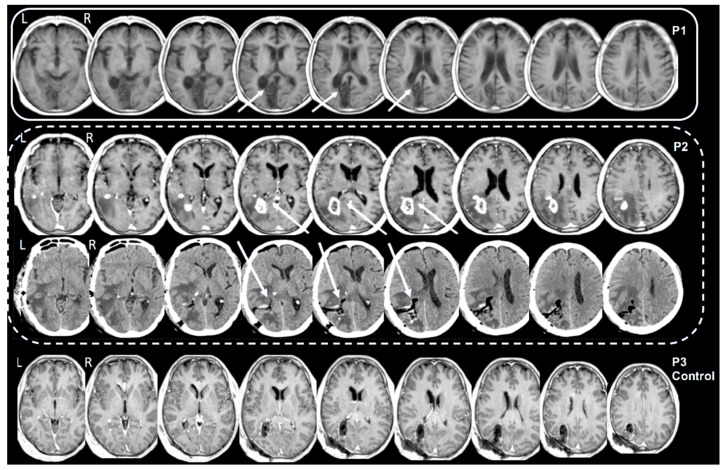
Patient 1 (P1) had a lesion that included the visual cortex and the forceps major continuing in the splenium of the corpus callosum. There was also a lesion in the left superior colliculus and part of the posterior left thalamus. In Patient 2 (P2, upper line), the MRI brain images pre-surgery, showed a large left posterior temporal and parieto-occipital neoplasm including the optic radiation, but the forceps major fibers were intact. Patient 2 (P2 lower line), the CT brain scan post-surgery showed the resection of the neoplasm including the optic radiation and the forceps major. The arrows indicate the brain region of the forceps major. For Patient 3 (P3 Control), the MRI brain images present a left posterior temporal and parieto-occipital resection of the neoplasm including the optic radiation, sparing the forceps major fibers. Note that none of the patients had lesions in the right hemisphere. L: left hemisphere; R: right hemisphere.

T1 weighted magnetic resonance imaging (MRI) clinical scans for P1 and P3 were acquired, respectively, 127 days after the injury and 1 day after surgery. For P2, a T1 weighted MRI scan was acquired the day of admission to the hospital and a computed tomography (CT) scan one day after surgery. The MRI and the CT brain volumes were separately transformed into the Average MNI Brain Space [20] using the interactive graphics software program Register (http://www.bic.mni.mcgill.ca/ServicesSoftwareVisualization/Register; accessed on 5 May 2021) to identify by visual inspection and compare the lesion sites accurately [21] (Figure 1). The assessment by visual inspection of the location of the lesions was based on atlases of the human brain [20,22].

### 2.2. Neglect Testing

After the examiner had explained to the patient what should be done either verbally or by showing her/him how to perform each task, the patient had to demonstrate that she/he understood what was required by either rephrasing the examiner’s request or showing the examiner how a specific task should be done. In addition, the patients were asked to repeat single words (i.e., 10 single words) and simple sentences (i.e., 3 sentences) and they obtained a perfect score, ensuring that they could hold and reproduce verbal information. Finally, they were verbally asked to show how to use simple objects without the objects (e.g., show me how to use a hammer), as well as to show when blindfolded how to use an object that they had in their hands (e.g., a pair of scissors that was placed in their hand). The patients were able to perform these tests without problems, which ensured that they understood and could carry out instructions.

**Figure 2:**
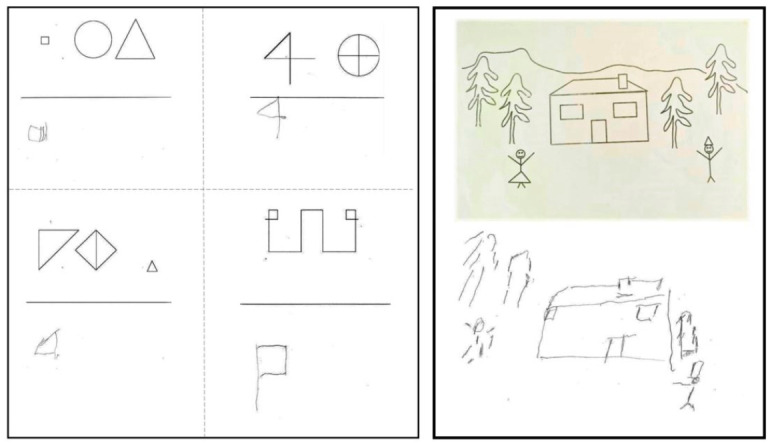
Samples of the copy of figures performed by the patients: (**Left**) panel shows the copy of simple figures performed by P1. She drew only the figure on the extreme left of her space. (**Right**) panel shows the copy of a figure drawn by P2. He did not draw the tree on the extreme part of the right space, the right arm of the person-figure in his right space, and a little part of the roof at its extreme right side.

*Visuo-spatial neglect:* Visuo-spatial neglect for the peri-personal space was assessed with the following tests: The Line Bisection task (line lengths of 100 and 200 mm), with five trials administered per line [23]; the Line Cancellation task [24]; the Letter Cancellation task [25] for P1, and a comparable Symbol Cancellation task (similar to that used by Vallar [26]) for P2 because of his alexia, were employed. Visuo-spatial neglect for the extra-personal space was assessed by asking the patients to indicate and possibly name pictures attached on the walls of the testing room (at more than 1 m distance). Note that the pictures presented to the patient were selected from those that he was able to name. Nevertheless, we accepted as a correct response the simple indication of the picture. Finally, a drawing task was used that required the copying of simple figures (see Figure 2).

*Personal-body neglect:* This was assessed using the Fluff Test [17]: while the patient was blindfolded and distracted by talking to her/him, six targets are attached on each leg, six targets on the right arm, and three targets are attached on the right and three on the left side of the torso (with respect to the sagittal body-midline). The patients are required to reach and remove with the left hand the 24 targets attached to their body while blindfolded. Thus, this test assesses personal-body neglect, and it is not a visual neglect test.

## 3. Results

### 3.1. Localization of the Brain Lesions

P1: The MRI scan revealed a lesion localised in the left hemisphere (see Figure 1, first line P1). There was clear damage within the forceps major region (i.e., the splenial fibers of the corpus callosum). The lesion extended to the striate and parastriate cortex, but part of the mesial extra-striate cortex above the cuneal sulcus was spared. There was also a lesion in the left superior colliculus and part of the posterior left thalamus. Note that there were no lesions in the right hemisphere.

P2: The pre-surgery MRI revealed a large left hemisphere posterior temporal and parieto-occipital neoplasm that included the optic radiation region, but the forceps major fibers were intact with an intense peripheral contrast-enhancing wall and a central hypointense necrotic area (Figure 1, P2 upper line). Clinical evidence of contralesional hemianopia and a tumoral lesion extensively infiltrating the left optic radiation suggested that the tumor resection should be performed through a parieto-temporal surgical approach. Fluorescence-guided resection of the lesion was performed after intravenous administration of sodium-fluorescein. The pegylated form of fluorescein showed minimal uptake in brain tissue and improved tumor-to-normal contrast by 38% [27]. The fluorescent tissue was resected including the white matter in the forceps major. The postoperative CT scan of the brain (Figure 1, P2 lower line) showed white matter removal adjacent to the posterior horn of the lateral ventricle, including the optic radiation, the forceps major splenial callosal fibers, the tapetum, and a small sector of the inferior longitudinal fasciculus. There were no lesions in the right hemisphere.

P3 (Control patient): The brain MRI scan revealed a lesion localised in the left hemisphere (see Figure 1, P3 Control, last line). There was clear damage to the optic radiation but there was sparing of the forceps major (i.e., the splenial fibers of the corpus callosum). There were no lesions in the right hemisphere.

### 3.2. Neglect Testing

**Figure 3:**
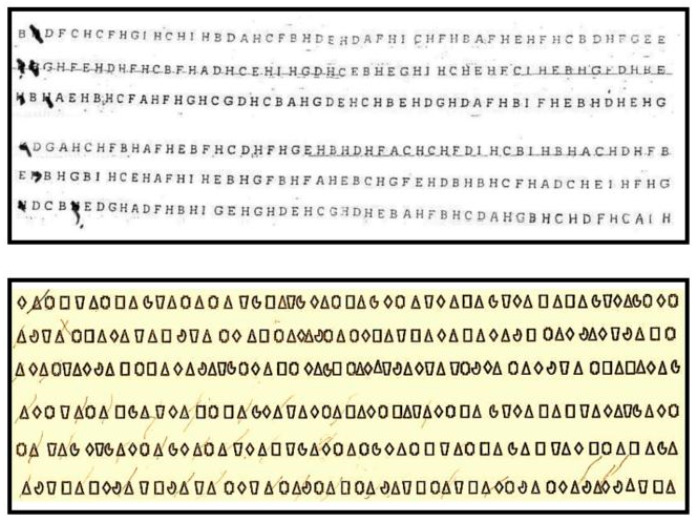
Cancellation Letter/Symbol tests. Upper panel shows the Letter Cancellation test performed by P1. She detected only 8 targets on her extreme left space. Lower panel shows the Symbol Cancellation test performed by P2. He detected 29 targets on his left space and 8 targets on his right space.

The performance scores of the patients on the tests used to assess neglect are presented in Table 1. The clinical testing in P1 and in P2 (post-surgery) revealed clear signs of right visuo-spatial neglect in both the peripersonal and extrapersonal spaces, but did not affect the personal-body space. Note that for P2, we adapted the Letter Cancellation test using symbols instead of letters because of the alexia exhibited by this patient. We systematically substituted the letter H with a black triangle, the letter F with a square, etc., from the Letter Cancellation test. Note that P1 had evidence of severe neglect on the right side since she cancelled only letters on her extreme left side. P2 also exhibited clear neglect on the right side since he only cancelled 8 out of the 51 symbols on the right side, while cancelling 29 out of 51 symbols on the left side. Thus, the neglect exhibited by P2 was milder compared with the neglect exhibited by P1 (see Figure 3). The patient P3, who did not have the splenial fibers removal performed, perfectly well on all the neglect tests.

## 4. Discussion

The present clinical study reports two rare cases of right visuo-spatial neglect resulting from damage to the PCA territory of the left hemisphere. One of these patients developed right hemianopia and right visuo-spatial neglect after an ischemic stroke of the PCA damaging the left primary visual cortex and the callosal splenial fibers. The other patient had a brain tumor in the posterior cerebral region and initially had only right hemianopia. However, after tumor resection that included the splenial fibers, the patient exhibited right visuo-spatial neglect. By contrast, P3, who had a clear damage of the optic radiation, but sparing of the forceps major (i.e., the splenial fibers of the corpus callosum), did present hemianopia but did not present visuo-spatial neglect. Thus, the hemianopia from a posterior left hemisphere lesion developed into unilateral neglect when the damage to the splenium of the corpus callosum disconnected the communication with the right hemisphere attentional systems. In line with anatomically equivalent cases in patients with right hemisphere brain damage, neglect was limited to the visual modality, and no neglect was present in personal-body space [7].

These data confirm that unilateral visual neglect cannot simply derive from lack of direct visual input to a specific hemisphere as a result of the geniculo-calcarine tract section or damage to the occipital visual cortex. In such cases, the damaged hemisphere that is no longer receiving direct visual input from the contralateral hemifield can still construct a cognitive representation of the contralesional visual space using visual information derived from fixations made in that space with the spared hemifield, i.e., with the spared hemisphere. Neglect emerges when visual information gathered by the intact hemisphere cannot reach the damaged “hemianopic” hemisphere because of additional callosal disconnection, as shown in an experimental lesion study in the macaque monkey [14]. More recently, Lunven and colleagues [11] have shown that white matter lesions involving the superior longitudinal fasciculi II and III disconnecting input from inferior parietal cortex to the frontal lobe result in neglect. They have also shown that damage to the splenium of the corpus callosum resulting in interhemispheric disconnection is related to the persistence of spatial neglect in the chronic phase.

The P1 and P2 cases examined in the present study confirm that contralesional neglect can be observed also after left, rather than right, PCA territory lesions. Nonetheless, it is worth noting that in a series of 45 patients with PCA territory lesions, Park and colleagues [12] found that, although right-side neglect following left PCA territory lesion is as frequent as left-side neglect following right PCA territory lesion, left-side neglect is generally more severe. These investigators concluded that this result is in agreement with the idea “*…that the right hemisphere is better able to attend to ipsilateral stimuli than is the left hemisphere*”.

A notable difference was present between P1 who suffered no alexia or anomia and P2 who had alexia and anomia. This difference indicates that in P1 the posterior language and word form areas in the left hemisphere were entirely spared (unlike in P2) and communication with posterior parietal and superior temporal areas of the right hemisphere occurred through the isthmus of the corpus callosum [28], thus compensating for the lack of inter-hemispheric interaction created by the splenial disconnection.

In P1, the presence of a lesion of the left superior colliculus and of the posterior thalamus may have contributed to the neglect. For example, it has been shown, in non-human primates, that a lesion of the superior colliculus causes inattention to stimuli in the affected visual field [29]. Recently, the same result has been reported in a single-case study in the human brain [30]. In the present study, the role of the combination of splenial disconnection with contralesional hemianopia in the production of visual neglect was supported by the findings in P2. Although suffering complete right homonymous hemianopia, P2 did not have signs of right-sided neglect before surgery. Clear right visuo-spatial neglect emerged only after surgical resection of the callosal fibers of the forceps major. The examination of the control P3, who did not have lesions of the splenial fibers, further supports the role of these fibers in the emergence of visuo-spatial neglect when hemianopia is present. These findings are consistent with those of earlier group studies [12] and single-case studies [7,15], suggesting that a unilateral lesion limited to the calcarine cortex or its input when it is combined with damage to the splenium of the corpus callosum can produce contralesional hemianopia combined with visuo-spatial neglect, without any involvement of collicular or thalamic structures. Sperber and colleagues [16] have recently pointed out that splenial damage alone does not necessarily result in hemispatial neglect. The present data demonstrate that splenial damage when combined with a unilateral lesion of the primary visual pathways producing hemianopia *does* yield hemispatial neglect. According to Sperber and colleagues [16], neglect in PCA cases would critically depend on the lesion involving perisylvian temporal-parietal areas. The present clinical case (P1) that had no perisylvian damage and neglect cases after PCA territory lesions with no involvement of perisylvian areas [7,12,15] do not support this hypothesis. In addition, Sperber and colleagues [16] did not investigate line bisection performance [20] but only performance on cancellation tasks. They speculate that a deficit on a line bisection task may be present in patients who have splenial lesion (but not in cancellation tasks that measure egocentric neglect). In patients P1 and P2 (post-surgery), we observed impaired performance on both line bisection and the cancellation task when damage involved the calcarine cortex (or input to it) in the left hemisphere in addition to damage to the splenium. Furthermore, in Sperber and colleagues [16], there was disconnection of input to the splenium (forceps major) and calcarine cortex lesions in the patients who presented impairment on the cancellation tasks (see [16] their Figure 1B, section z +16), although the authors emphasize the importance of more lateral damage (i.e., the border of the PCA and MCA territories). Finally, in our study, neglect was specific to the visual tasks and not to the Fluff test [17], which assesses personal space in the blindfolded condition. We believe that different networks underlie these different symptoms of the neglect syndrome. In the study by Sperber and colleagues [16], visual field testing was performed at bedside through neurological visual confrontation testing or via PC-based monitoring. The latter testing procedure might not be fully appropriate to ascertain that hemianopia was complete and that, therefore, no visual information could reach the lesioned hemisphere directly from the contralesional visual hemi-field.

## 5. Conclusions

The present investigation provides evidence that contralesional hemianopia resulting from damage of the geniculo-calcarine pathway can evolve into contralesional *hemianopia combined with visual-spatial neglect* when the splenial fibers of the corpus callosum are additionally damaged. Note that splenial damage does not only disconnect interhemispheric communication between the occipital cortical areas, but also communication from the adjacent parietal-occipital region, as shown by an examination of interhemispheric connectivity using diffusion tensor imaging in the human brain [28]. The finding from the present study that geniculo-calcarine damage combined with splenial damage results in *hemianopia with visuo-spatial neglect* highlights the behavioral consequences of the removal of tumoral tissue in the region of the splenium of the corpus callosum in hemianopic patients (see P2). An examination of interhemispheric connectivity using diffusion tensor imaging in these patients would have further supported the neuroanatomical evidence revealed by the clinical MRI and CT morphological brain images. Finally, awake surgery combined with behavioral evaluation is recommended to limit and control potential neuropsychological deficits caused by surgical intervention [2]. Nevertheless, lesions located in the parieto-occipital region may require a prone position in the operating theatre: such positioning increases the complexity of awake surgery because of possible difficulties in the management of the airways by anaesthesiologists, as a well as reducing the possibility of presenting visual stimuli and control for patient movement. In addition, such positioning would limit required performances on neuropsychological tests during awake surgery. Thus, the exploration of neuropsychological functions, such as visuo-spatial abilities during awake surgery, requires the expertise of specifically trained neuropsychologists, neurosurgeons, anaesthesiologists, and a specific setting in the operating room.

## Figures and Tables

**Table 1:** The clinical testing of P1 and P2 (post-surgery) revealed clear signs of right visuo-spatial neglect that was present in both the peripersonal and extrapersonal right half of space, but there were no signs of neglect in personal-body space. P3 performed correctly on all the neglect tests. Scores in bold indicate impaired performance. The sign “-” indicates a test that was not administered.

Neglect Testing		P1	P2		P3 (Control)		Cut-Off
			Pre-Surgery	Post-Surgery	Pre-Surgery	Post-Surgery	
Line Bisection [23] *(distance from the center of the line)*	100 mm	1	0	**8**	0	0	>3 mm
	200 mm	**28.6**	2	**53**	0	0	>6 mm
Line Cancellation [24]	Left	9/10	10/10	10/10	10/10	10/10	
	Right	0/11	11/11	10/11	11/11	11/11	
*(between-sides difference)*		**9**	0	**1**	0	0	>0
Letter or Symbol Cancellation [26]	Left	4/53	-	29/53	53/53	53/53	
	Right	0/51	-	8/51	51/51	51/51	
*(between-sides difference)*		**4**		**21**		0	>3
Room Inspection	Left	5/5	-	5/5	5/5	5/5	
	Right	2/5	-	2/5	5/5	5/5	
*(between-sides difference)*		**3**		**3**		0	>0
Personal-body, Fluff test [17]	Left	9/9	-	9/9	-	9/9	
	Right	13/15	-	13/15	-	15/15	<12

## Data Availability

Upon reasonable request, neuroimaging data and scripts can be made available to interested parties by contacting the corresponding authors.
